# Evaluation of occlusal force changes in orthognathic surgery using force-sensing sensors in 3 years of follow-up

**DOI:** 10.1097/MS9.0000000000002386

**Published:** 2024-07-17

**Authors:** Fernando Duarte, João Neves Silva, Carina Ramos, Colin Hopper

**Affiliations:** a Eastman Dental Institute, University College of London; b UCL Eastman Dental Institute, London, UK; c Universitá Cattolica del Sacro Cuore, Rome, Italy; d Clitrofa, Trofa; e Instituto Superior de Saúde; f Instituto Universitário de Ciências da Saúde, Portugal

**Keywords:** force-sensing sensors, occlusal force, orthognathic surgery

## Abstract

**Purpose:**

The aim of this study was to test a prototype device called occlusal force diagnostic system in relation to occlusal force adaptation following orthognathic surgery.

**Methods:**

Retrospective study of 10 patients scheduled for a bimaxillary osteotomy involving a combination of maxillary Le Fort I impaction procedure coupled with a sagittal split advancement of the mandible; in a 3 years follow-up period.

**Results:**

The selection of examiner is not a variable that affects the occlusal force (N) measured by FSS sensors in any of the experimental conditions tested. The sensor position and the surgery recovery time affect the occlusal force irrespective of the examiner selection and/or the surgery recovery time.

**Conclusion:**

The piezoelectric sensors used in the present study have shown high reliability and validity of measurement. The surgery recovery time impacts the occlusal force (N), with a 50% increase in occlusal force (N) measured after 6 months post-surgery, with the value keeping stable at 36 months. This suggests that the patient is only fully recovered from the functional point-of-view at 6 months, having from that point on an improved and stable masticatory function.

## Introduction

One of the main purposes of orthognathic treatment in patients with a dentofacial deformity is to improve masticatory function as well as aesthetics^1–3^. Numerous studies have documented masticatory function, for example bite force, occlusal contact and masticatory efficiency, in patients with mandibular prognathism before and after orthognathic surgery^4–13^; but few reports compared the results with those in controls with normal occlusion^1,3,6–9,12,13^. There have also been few studies that involved the evaluation of these parameters at the initial medical consultation for patients undergoing orthognathic surgery^14,15^. No reports were found that simultaneously evaluated the relationships between bite force, occlusal contact and masticatory efficiency in patients with mandibular prognathism and in controls with normal occlusion.

Previously, changes in bite force and occlusal contact before and after orthognathic surgery were investigated and presented using the T-Scan system (Tekscan, USA)^3^. This system is convenient and simple but is poor in regard to reproducibility and quantification.

Another method for occlusal analysis, the Dental Prescale system (Fuji Photo Film Co.), has been developed. This is a computerised system intended to assist occlusal analysis by providing information as to the magnitude of the bite force and the distribution of occlusal contacts. The system is capable of simultaneously measuring these parameters for teeth separated by less than 10 mm and has the potential for research in centric occlusion. It is a horseshoe-shaped thin film that consists of two layers: a layer of microcapsules containing colour-forming materials and a layer of colour-developing materials. The colour-developing materials, producing a red colour in the contact area when a force is generated, absorb the released colour-forming materials. The Dental Prescale system has already been used for analysing occlusion in dentures^16,17^ dental implants^18^ and orthognathic surgery^2,8^.

Many methods for the quantitative measurement of masticatory efficiency have been introduced, but none stands out as ideal. Spectrophotometric methods for the evaluation of masticatory efficiency have been reported, involving measurement of the absorbance of adenosine triphosphate (ATP) granules^6,7,12^. This technique shows both accuracy and reproductibility, but it has an high cost and complexity. A chewing-gum system has been developed for the estimation of masticatory function by the Meiji Chewing Gum Corporation. It utilises a phloxine–sodium bicarbonate reaction and measures a chromatic coordinate as an indicator. This low-adhesive colour-developing chewing-gum system has already been used for analysing the masticatory function of dental implants^19^ and dentures^20^, but it does not allow quantitative determination^21^.

It is now accepted that because there is no single method of assessing masticatory function, several measures should be taken, and whenever possible, simultaneously. This pilot investigation is designed to apply newly developed and more sophisticated methods of measuring muscle function to a situation where adaptation of muscle is pivotal to the success of a therapeutic approach.

## Materials and methods

The stability of orthognathic surgery is related to the adaptation of the masseter muscle, recurrence is a constant in the post-surgical course most frequently within 6 months from the operation. The aim of this study was to test a prototype device called occlusal force diagnostic system that will provide information in relation to occlusal force adaptation following orthognathic surgery.

The FSS sensors provide precise reliable force-sensing performance in a compact commercial-grade package. The sensor features a proven sensing technology that uses a specialized piezoresistive micromachined silicon-sensing element. The low-power, unamplified, uncompensated wheatstone bridge circuit design provides inherently stable mV outputs over the force range^22^.

**Figure 1:**
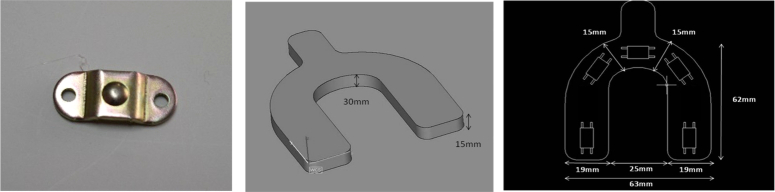
FSS sensor image, arch dimensions and sensors distribution.

Force sensors operate on the principle that the resistance of silicon-implanted piezoresistors will increase when the resistors flex under any applied force. The sensor concentrates force from the applications, through the stainless-steel ball, directly to the silicon-sensing element. The amount of resistance changes in proportion to the amount of force being applied. This change in circuit resistance results in a corresponding mV output level change (Fig. 1)^22,23^.

In this prototype device called occlusal force diagnostic system, five sensors were distributed in the following order with the readings in kilograms. Sensor A: right maxillary second pre-molar and right maxillary first molar between 1st and 4th quadrants; Sensor B: right maxillary canine and right maxillary first pre-molar between 1st and 4th quadrants; Sensor C: right and left maxillary central incisors and right and left maxillary lateral incisors area; Sensor D: left maxillary second pre-molar and left maxillary first molar between 2nd and 3rd quadrants, and finally Sensor E: left maxillary canine and left maxillary first pre-molar between 2nd and 3rd quadrants (Fig. 1).

The dental arch in a horseshoe-shaped form was built by a superior and an inferior 3 mm height metal foil covered by a hard resin, with the following intra-oral measures: 63 mm total width, 62 mm total length, 15 mm width in the anterior occlusal contact area, 19 mm width in the posterior occlusal contact area, 30 mm anterior height and 15 mm posterior height. The dental arch dimensions were based on the majority of the dental arches studied during the improvement process (Fig. 1).

The present study is a retrospective study with quantitative methodology. A study group of 10 patients attending the combined orthodontic/orthognathic surgery clinic at the Clitrofa, Centro Médico, Dentário e Cirúrgico, in Trofa, Portugal was selected to the present study by a convenience non-probability sampling method. All the selected patients were scheduled for a bimaxillary osteotomy involving a combination of maxillary Le Fort I impaction procedure coupled with a sagittal split advancement of the mandible were select to form the study group.

The Occlusal Force Diagnostic System was placed between the upper and lower dental arch, and the subjects were instructed to bite as forcefully as possible for about 3 seconds. The values were registered by two different observers (F and C) in different moments: (T0) - before surgery, (T1) - 10 min after surgery, (T2) - 1 month after surgery, (T3) - 6 months after surgery and (T4) - 36 months after surgery.

### Statistical analysis

IBM SPSS, version 25, was used to analyse the data obtained. Exploratory data analysis was performed by Kolmogorov–Smirnov ( D ) test to assess the normality of the frequency distributions and by Levene test ( L ) to assess the variance homogeneity of the variables.

Descriptive statistics of the study variables was performed by determination of mode and frequencies (nominal variables), median and interquartile range (ordinal variables), and arithmetic mean and standard deviation (numerical variables). Bar graphs were also added to facilitate data description and results interpretation.

Inferential statistics was used to compare examiner selection (paired two-tailed Student’s *t*-test), sensor position (Repeated Measures ANOVA) and surgery recovery time (Repeated Measures ANOVA). Where the requirements for parametric statistical analysis were not met, the inferential tests were replaced, respectively, by Wilcoxon, Friedman and Friedman tests.

**Figure 2:**
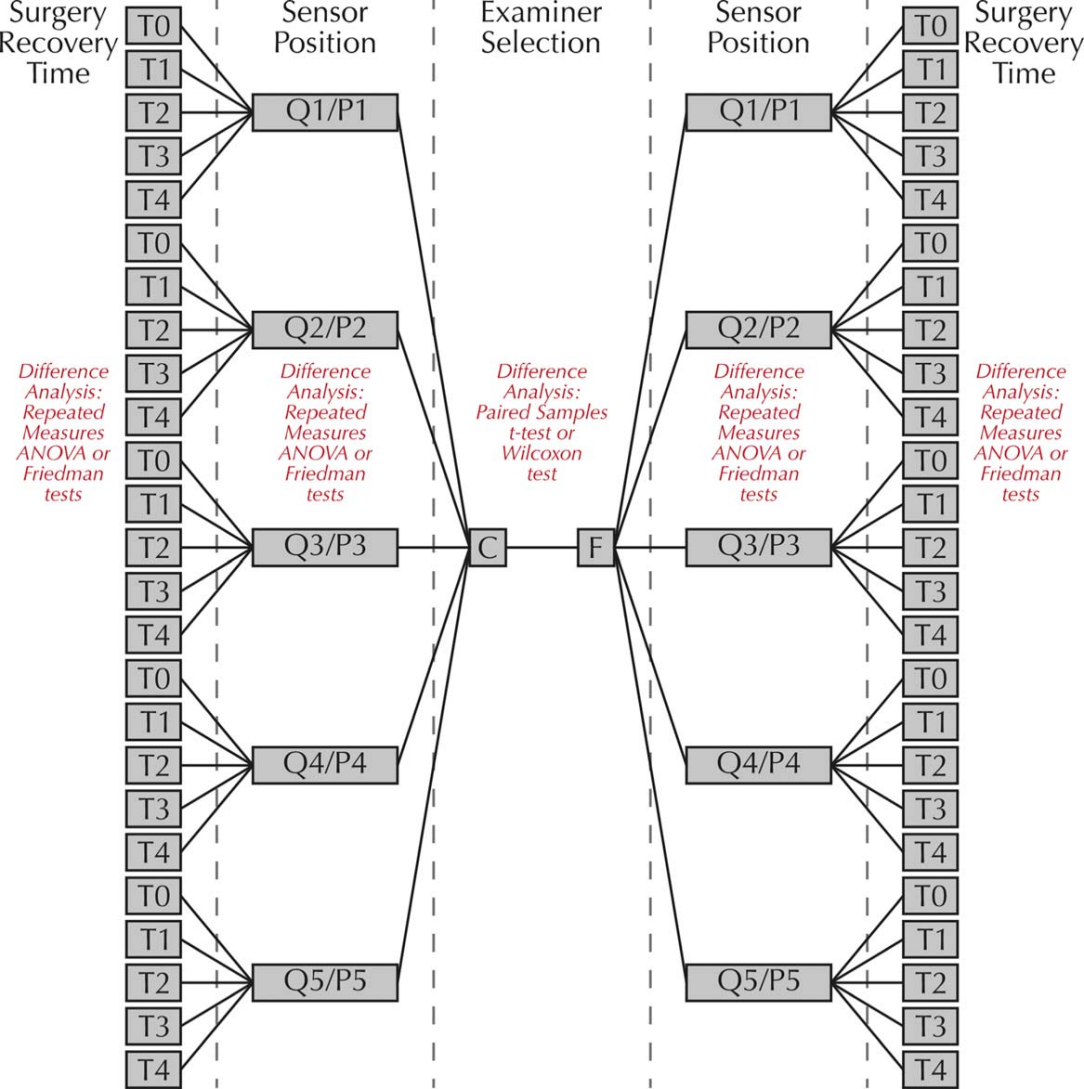
Experimental design used in the present study to evaluate the effect of examiner selection (F or C), sensor position (Q1/P1, Q2/P2, Q3/P3, Q4/P4 or Q5/P5) and surgery recovery time (T0 - before surgery, T1 - 10 min after surgery, T2 - 1 month after surgery, T3 - 6 months after surgery, or T4 - 36 months after surgery) on the occlusal force (N) measured by FSS sensors in the 10 patients of the sample.

The experimental design used in this study is depicted in Figure 2 and comprises 3 separate researches:

1. Research A, which investigated the effect of examiner selection on the occlusal force (N) measured by FSS sensors;
2. Research B, which investigated the effect of sensor position on the occlusal force (N) measured by FSS sensors;
3. Research C, which investigated the effect of surgery recovery time on the occlusal force (N) measured by FSS sensors.

Where statistically significant differences were found by repeated measures ANOVA tests, the multiple-comparison Post-Hoc Bonferroni or Gabriel tests were performed to identify the pairs of categories were the statistically significant differences were located (Fig. 2).

Three thresholds of statistical significance (α level) were considered throughout the present study: *p* values below 0.05 (*) were considered statistically significant; *p* values below 0.01 (*) were considered highly statistically significant, and *p* values below 0.001 (*) were considered very highly statistically significant. The lack of statistical significance was designated as non-significant (ns).

## Results

In order to make the presentation of results easier to understand; they were subdivided into four items, as follows: data exploratory analysis, the effect of examiner selection, the effect of sensor position and effect of surgery recovery time.

### Data exploratory analysis

Kolmogorov–Smirnov ( D ) and Levene ( L ) assumption tests have revealed that the study variables do not comply the minimum requirements for an inferential parametric analysis (normality of frequency distributions and variance homogeneity), thus meaning that the effects of examiner selection, sensor position and surgery recovery time on the occlusal force (N) measured by FSS sensors will be analysed by the differences tests of Wilcoxon ( U ), Friedman ( H ) and Friedman ( H ), respectively presented in Table 1.

**Table 1:** Data exploratory analysis

Study variables	Central tendency measures	Dispersion measures	Kolmogorov–Smirnov test ( D ); *P*	Levene test ( L ); *P*
Examiner selection	Mode: C, F	Frequencies: C (50.0%); F (50.0%)	D : 0.339 Pvalue : 0.000***	L : 0.000 Pvalue : 0.989
Sensor position	Mode: Q1/P1; Q2/P2; Q3/P3 ; Q4/P4 ; Q5/P5	Frequencies: Q1/P1 (20.0%); Q2/P2 (20.0%); Q3/P3 (20.0%); Q4/P4 (20.0%); Q5/P5 (20.0%)	D : 0.158 Pvalue : 0.003***	L : 29.295 Pvalue : 0.000***
Surgery recovery time	Median: 3 (T2)	Interquartile Range: 2	D : 0.158 Pvalue : 0.003***	L : 0.911 Pvalue : 0.466
Occlusal force (N)	Mean: 46.87	SD: 19.56	NA	NA

NA, not applicable.

* Significant statistical difference to an alpha level of 0.05.

** Highly significant statistical difference to an alpha level of 0.01.

*** Very highly significant statistical difference to an alpha level of 0.001.

### Research a: effect of examiner selection on the occlusal force (n) measured by FSS sensors

**Figure 3:**
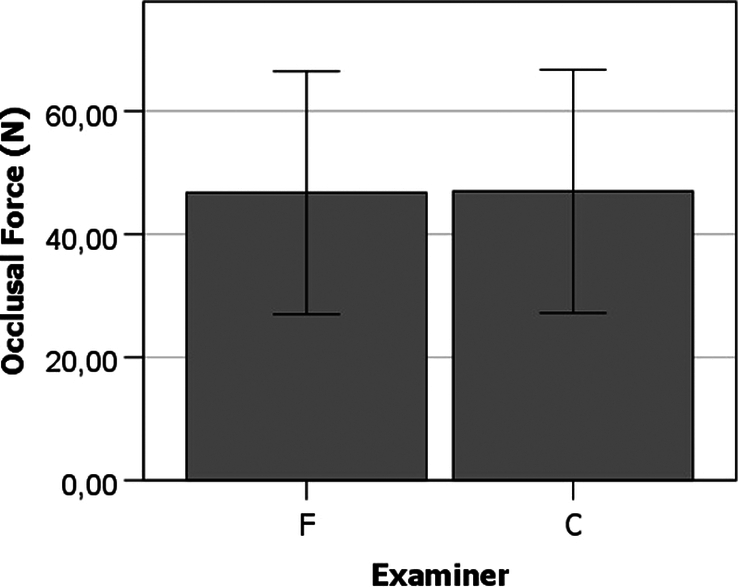
Effect of examiner selection on the occlusal force (N) measured by FSS sensors. Error bars represent standard deviation.

Figure 3 shows the similarity of occlusal force (N) measurements made by examiners F and C. The relatively high standard deviation of the measures depicted in Figure 3 arises from the fact that the examiners have been compared in different experimental conditions (sensors positions and surgery recovery times), which are in the graphic are presented in the same group of values.

Wilcoxon ( U ) tests have revealed the general absence of significant statistical differences between examiners F and C regarding the occlusal force (N) measured by FSS sensors in the 10 patients of the sample, in the different experimental conditions tested, presented in Table 2.

**Table 2:** Effect of examiner selection on the occlusal force (N) measured by FSS sensors (Wilcoxon ( U ) test)

Experimental conditions	Wilcoxon ( U )	*P*
F vs. C, Q1/P1, T0	−0.135	0.893
F vs. C, Q1/P1, T1	−10.763	0.078
F vs. C, Q1/P1, T2	−0.355	0.723
F vs. C, Q1/P1, T3	−0.271	0.786
F vs. C, Q1/P1, T4	−0.577	0.564
F vs. C, Q2/P2, T0	−10.633	0.102
F vs. C, Q2/P2, T1	−0.061	0.952
F vs. C, Q2/P2, T2	0.000	1.000
F vs. C, Q2/P2, T3	−0.632	0.527
F vs. C, Q2/P2, T4	−10.633	0.102
F vs. C, Q3/P3, T0	0.000	1.000
F vs. C, Q3/P3, T1	0.000	1.000
F vs. C, Q3/P3, T2	0.000	1.000
F vs. C, Q3/P3, T3	−20.121	0.034*
F vs. C, Q3/P3, T4	0.000	1.000
F vs. C, Q4/P4, T0	0.000	1.000
F vs. C, Q4/P4, T1	−0.137	0.891
F vs. C, Q4/P4, T2	−10.550	0.121
F vs. C, Q4/P4, T3	0.000	1.000
F vs. C, Q4/P4, T4	−0.816	0.414
F vs. C, Q5/P5, T0	−10.236	0.216
F vs. C, Q5/P5, T1	−0.141	0.888
F vs. C, Q5/P5, T2	0.000	1.000
F vs. C, Q5/P5, T3	−0.302	0.763
F vs. C, Q5/P5, T4	−10.633	0.102

* Significant statistical difference to an alpha level of 0.05.

** Highly significant statistical difference to an alpha level of 0.01.

*** Very highly significant statistical difference to an alpha level of 0.001.

### Research b: effect of sensor position on the occlusal force (n) measured by FSS sensors

**Figure 4:**
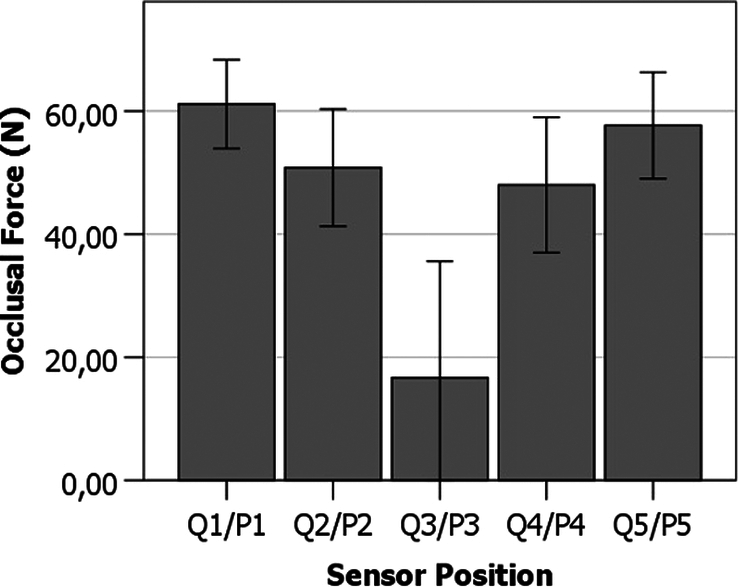
Effect of sensor position on the occlusal force (N) measured by FSS sensors. Error bars represent standard deviation.

Figure 4 shows the variation of occlusal force (N) measurements made with the different sensor positions. Results indicate a decrease in occlusal force (N) as the sensor position is placed closer to the temporomandibular joint. Additionally, pairs of sensors placed on the same left/right plan (pairs P2/P4 and P1/P5) detect identical occlusal forces (N), which show a homogeneous bite force in the frontal plane of the patients that compose the sample. The relatively high standard deviation of the measures depicted in Figure 4 arises from the fact that the sensor positions have been compared in different experimental conditions (examiner selection and surgery recovery times), which are in the graphic are presented in the same group of values.

Friedman ( H ) tests have revealed the presence of presence of very highly significant statistical differences between the different sensor positions (Q1/P1, Q2/P2, Q3/P3, Q4/P4 and Q5/P5) regarding the occlusal force (N) measured by FSS sensors in the 10 patients of the sample, in the different experimental conditions tested, presented in Table 3.

**Table 3:** Effect of sensor position on the occlusal force (N) measured by FSS sensors (Friedman ( H ) test)

Experimental conditions	Friedman ( H )	*P*
Q1/P1 vs. Q2/P2 vs. Q3/P3 vs. Q4/P4 vs. Q5/P5, F, T0	21.340	0.000***
Q1/P1 vs. Q2/P2 vs. Q3/P3 vs. Q4/P4 vs. Q5/P5, F, T1	20.551	0.000***
Q1/P1 vs. Q2/P2 vs. Q3/P3 vs. Q4/P4 vs. Q5/P5, F, T2	20.447	0.000***
Q1/P1 vs. Q2/P2 vs. Q3/P3 vs. Q4/P4 vs. Q5/P5, F, T3	26.330	0.000***
Q1/P1 vs. Q2/P2 vs. Q3/P3 vs. Q4/P4 vs. Q5/P5, F, T4	25.980	0.000***
Q1/P1 vs. Q2/P2 vs. Q3/P3 vs. Q4/P4 vs. Q5/P5, C, T0	20.975	0.000***
Q1/P1 vs. Q2/P2 vs. Q3/P3 vs. Q4/P4 vs. Q5/P5, C, T1	20.469	0.000***
Q1/P1 vs. Q2/P2 vs. Q3/P3 vs. Q4/P4 vs. Q5/P5, C, T2	21.082	0.000***
Q1/P1 vs. Q2/P2 vs. Q3/P3 vs. Q4/P4 vs. Q5/P5, C, T3	24.914	0.000***
Q1/P1 vs. Q2/P2 vs. Q3/P3 vs. Q4/P4 vs. Q5/P5, C, T4	25.629	0.000***

* Significant statistical difference to an alpha level of 0.05.

** Highly significant statistical difference to an alpha level of 0.01.

*** Very highly significant statistical difference to an alpha level of 0.001.

**Figure 5:**
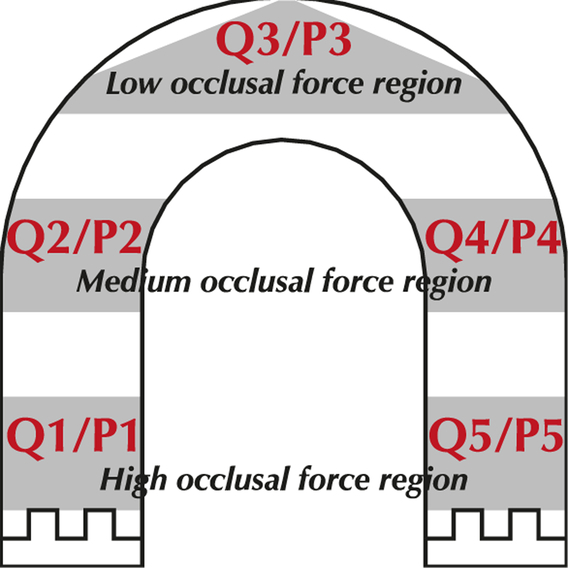
Three-pressure region model for dental occlusion.

This variation in occlusal force (N) measured by FSS sensors due to the sensor position was already expected and is related to the dynamics of the human masticatory function, where the temporomandibular joint (biomechanically characterised as a third-class lever (inter-potent), the different masticatory muscles (active and passive) and the complex jaw movements play an important role to mandible occlusion^24^. From the occlusal force (N) measurement point-of-view, the higher the distance between the sensor position and the temporomandibular joint, the higher the occlusal force (N) measured, as suggested by the following illustrative model in Figure 5:

### Research c: effect of surgery recovery time on the occlusal force (n) measured by FSS sensors

**Figure 6:**
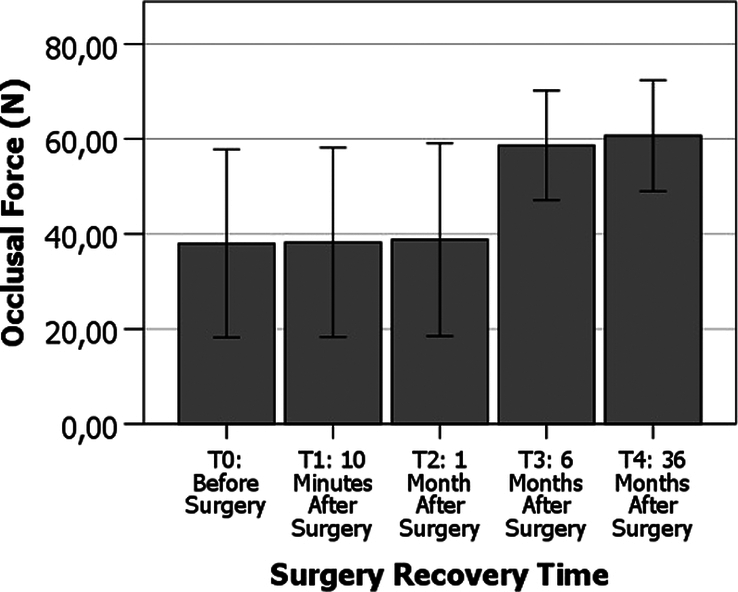
Effect of surgery recovery time on the occlusal force (N) measured by FSS sensors. Error bars represent standard deviation.

Figure 6 shows the variation of occlusal force (N) measurements at different surgery recovery times. One of the most innovative aspects of the present study is that the follow-up period of the patients has been extended and reported until 36 months, thus allowing a more complete view of the patient’s recovery process, as viewed by the masticatory force generated by the mandible. The data suggest a 50% increase in occlusal force (N) from 1 to 6 months in the patients, with the value stabilizing from that point until 36 months. It now becomes apparent that the increase in occlusal force produced by the surgery has a long-term stability, which shows the success of the clinical approach and the improvement of the patient’s life quality.

‘Friedman ( H ) tests have revealed the presence of very highly significant statistical differences between the different surgery recovery times (T0, T1, T2, T3 and T4) regarding the occlusal force (N) measured by FSS sensors in the 10 patients of the sample, in the different experimental conditions tested; shown in Table 4.

**Table 4:** Effect of surgery recovery time on the occlusal force (N) measured by FSS sensors (Friedman ( H ) test)

Experimental conditions	Friedman ( H )	*P*
T0 vs. T1 vs. T2 vs. T3 vs. T4, F, Q1/P1	23.505	0.000***
T0 vs. T1 vs. T2 vs. T3 vs. T4, C, Q1/P1	21.751	0.000***
T0 vs. T1 vs. T2 vs. T3 vs. T4, F, Q2/P2	19.176	0.001***
T0 vs. T1 vs. T2 vs. T3 vs. T4, C, Q2/P2	19.311	0.001***
T0 vs. T1 vs. T2 vs. T3 vs. T4, F, Q3/P3	37.739	0.000***
T0 vs. T1 vs. T2 vs. T3 vs. T4, C, Q3/P3	38.717	0.000***
T0 vs. T1 vs. T2 vs. T3 vs. T4, F, Q4/P4	13.587	0.009**
T0 vs. T1 vs. T2 vs. T3 vs. T4, C, Q4/P4	12.645	0.013*
T0 vs. T1 vs. T2 vs. T3 vs. T4, F, Q5/P5	9.040	0.060
T0 vs. T1 vs. T2 vs. T3 vs. T4, C, Q5/P5	9.822	0.044*

* Significant statistical difference to an alpha level of 0.05.

** Highly significant statistical difference to an alpha level of 0.01.

*** Very highly significant statistical difference to an alpha level of 0.001.

## Discussion

Relapse is a potential risk after orthognathic surgery^25^. The incidence of relapse after orthognathic surgery has been the subject of extensive investigation in recent years, and it is a continuous process that needs to be assessed in the present and in the future^26^. Compared to the general population, the risk of relapse is greater in cleft lip and palate (CL/P) patients due to more risk factors^27^. The association between CL/P and a higher likelihood of relapse is well acknowledged, even though additional causes are not fully understood^27^. In a study of da Silva *et al*.^27^, considering previous to surgery identical overjet values and degree of maxillary advancement in the groups with and without cleft, it was found that patients who had CL/P had an average relapse of 1248 cm, more than patients who did not have CL/P.

The first few days following surgery are quite challenging for the patients. Following the orthognathic surgery treatment, the postoperative healing period might take weeks or months^28^. The detection of relapse and its complex effect can be minimised by identifying their causes^28^.

The relationship between occlusal and bite forces before and after orthognathic surgery has been extensively studied^29,30^. Most of these studies have evaluated bite force using different approaches and have reported varying outcomes^30^.

In this study the selection of examiner was not a variable that affects the occlusal force measured by FSS sensors in any of the experimental conditions tested. The sensor position affects the occlusal force, irrespective of the examiner selection and/or the surgery recovery time.

In a recent systematic review and meta-analysis, with a protocol developed in accordance with the Preferred Reporting Items for Systematic Reviews and Meta-Analysis Protocols (PRISMA-P), a search strategy was considered that resulted in 978 articles. The authors presented the following conclusions: occlusal strength increased after orthognathic surgery, although not to the level of the control group; however, maximum bite force remained unchanged. Immediately after orthognathic surgery, chewing and swallowing forces increased. Significant reductions in postoperative occlusal contact pressure areas were also observed^30^. The results of this work are in agreement with our study in terms of occlusal force.

Throckmorton and Ellis^31^ presented a study comparing sagittal split ramus osteotomy with and without Le Fort I osteotomy. This study evaluated two variables: lip closing force and occlusal force. The occlusal force was evaluated using dental prescale and occluzer, which was connected to a computer interface; and they inferred that occlusal force improved postoperatively after orthognathic surgery^31^.

In a publication of Harada *et al*.^32^ performing Le Fort I and bilateral sagittal split osteotomy using occluzer, reported improved bite force postoperatively after orthognathic surgery. These authors were in agreement to the study published by Throckmorton and Ellis, which opined that increased bite force after orthognathic surgery could be due to a difference in the morphology of dentoskeletal structure^31,32^.

In our study the surgery recovery time affects the occlusal force measured by FSS sensors, irrespective of the examiner selection and/or the sensor position. The duration of recovery of bite force has not been consistent among various studies^30^. The recovery was assessed in most of studies using variables including asymmetry, EMG activity of temporalis and masseter muscle attachments, dentoskeletal abnormalities, and lip function tests. In all of these studies, the recordings were performed preoperatively and at 1, 3, 6, and 12 months postoperatively^30^.

In terms of the advantages of the device presented, we can highlight the high repeatability presented as well as the sensitivity in measuring pre and postoperative occlusal changes. From the point of view of limitations, we must mention the small number of clinical cases in which it was tested, which does not allow us to assess strong resolutions.

## Conclusion

The piezoelectric sensors used in the present study have shown high reliability and validity of measurement. The selection of the examiner does not affect the measurement of occlusal force (N), which shows good inter-examiner reliability. The sensor position influences the occlusal force (N) that is measured, with the increase on sensor/temporomandibular joint distance increasing the occlusal force (N) measured, which is related to the complex dynamics of the human masticatory system. The surgery recovery time impacts the occlusal force (N), with a 50% increase in occlusal force (N) measured after 6 months post-surgery, with the value keeping stable at 36 months. This suggests that the patient is only fully recovered from the functional point-of-view at 6 months, having from that point on an improved and stable masticatory function.

## Ethical approval

This project has approval by the Joint Research & Ethics Committee of UCL Hospitals NHS Trust, Reference No.03/E012.

## Consent

Written informed consent was obtained from the patients for publication of this study and accompanying images. A copy of the written consent is available for review by the Editor-in-Chief of this journal on request.

## Source of funding

The authors received no financial support for the research, authorship, and/or publication of this article.

## Author contribution

F.D., J.N.S., and C.R. read and wrote the manuscript. F.D. and C.R. were responsible for conducting surgeries. F.D. and J.N.S. were responsible for the data collection. F.D. designed and wrote the entire article. C.H. was responsible for the final revision of the manuscript. All authors read and approved the final manuscript.

## Conflicts of interest disclosure

The authors declare that they have no competing interests.

## Research registration unique identifying number (UIN)

This project is covered by the UCL Data Protection Registration Reference No. Z6364106, Section 19, Research: Health Research.

## Guarantor

Fernando Duarte.

## Data availability statement

No datasets were generated or analysed during the current study.

## Provenance and peer review

Not commissioned, externally peer-reviewed.
